# Gratitude, Hope, Mindfulness and Personal-Growth Initiative: Buffers or Risk Factors for Problem Gambling?

**DOI:** 10.1371/journal.pone.0083889

**Published:** 2014-02-11

**Authors:** Jasmine M. Y. Loo, Jung-Shun Tsai, Namrata Raylu, Tian P. S. Oei

**Affiliations:** 1 Jeffrey Cheah School of Medicine and Health Sciences, Monash University Malaysia, Sunway, Selangor Darul Ehsan, Malaysia; 2 Yuhing Junior College of Health Care and Management, Kaohsiung, Taiwan; 3 School of Psychology, The University of Queensland, Brisbane, Queensland, Australia; The University of Hong Kong, Hong Kong

## Abstract

The majority of prevention and intervention research in problem gambling (PG) has focused on identifying negative risk factors. However, not all at-risk individuals go on to develop anticipated disorders and many thrive in spite of them. In healthcare settings, PG and other disorders are typically conceptualized from the biomedical perspective that frame disorders as something negative residing within the individual and reduction in negativity is seen as success. Indeed, this problem-focused conceptualization may be adequate in many cases as reducing PG behaviour is undoubtedly an important outcome, but the focus on negativity alone is too narrow to capture the complexity of human behaviour. Hence, this study attempts to bridge the gap in literature by providing an evaluation of the predictive ability of the positive dispositions on problem gambling severity, gambling-related cognitions, and gambling urges. The positive psychological dispositions examined were curiosity, gratitude, hope, personal growth initiative, and mindfulness. Participants consisted of 801 Taiwanese Chinese students and community individuals (Mean age = 25.36 years). Higher levels of gratitude and hope have been found to predict lower PG, gambling-related cognitions, or gambling urges. Meanwhile, higher mindfulness predicted lower PG, but only among Chinese males. However, lower personal growth initiative predicted lower PG, gambling-related cognitions, and gambling urges. These analyses have small to medium effect sizes with significant predictions. Findings of this study have essential implications in understanding and treating Chinese problem gamblers. These positive dispositions should be addressed by mental health professionals in preventative and treatment programs among Chinese individuals. Further implications and suggestions for future research are discussed.

## Introduction

Gambling revenues have increased over the years in countries such as the United States, Australia, Taiwan, Singapore, and Macao [3]. It is reasonable to argue that the percentage of individuals involved in gambling activities will also increase, as gambling revenues increase. To date, estimates of reported gambling participation varies from 26.6% [4], [5] to 92% [6] in Chinese samples from Canada and New Zealand respectively. In Australia, approximately 16,000 adult Queenslanders are classified as problem gamblers (PGs) and 57,000 individuals are classified in the moderate risk of gambling group [7]. Generally, gambling can be termed as an act of risking a sum of money on the outcome of a game or event, which is determined by chance [8]. When gamblers win or lose, a range of readily perceptible cognitions, emotions, and behaviours are evoked. In turn, these factors may drive a vicious cycle of excessive gambling with detrimental consequences such as financial debt, work and health issues, and strained relationships [9], [10]. Despite the alarming rate of PG, it has been argued that many PGs do recover after seeking treatment and they would in turn assist other PGs struggling with gambling addiction [11].

The majority of prevention and intervention research in problem gambling (PG) focused on identifying negative risk factors. However, individual differences have been identified that buffer the risks of developing anticipated disorders, and many individuals flourish despite having been diagnosed with being at-risk [1]. In healthcare settings, PG and other disorders are typically conceptualized from the biomedical perspective that frames disorders as something negative residing within the individual and reduction in negativity is seen as success [2]. Indeed, this problem-focused conceptualization may be adequate in many cases as reducing PG behaviour is undoubtedly an important outcome, but the focus on the negative alone is too narrow to capture the complexity of human behaviour. It is also important to focus on positive traits such as hope and mindfulness so that individuals can experience an improvement in overall quality of life alongside cessation of PG. As such, the individual is less vulnerable to relapse or recurrence [12], [13]. By reframing disorders to include the absence of positive traits and protective factors, in addition to our knowledge of the influence of negative characteristics, we will gain a more comprehensive understanding of both positive and negative factors that affect the development of gambling problems and be better prepared in tailoring effective intervention strategies.

Attaining and maintaining psychological health and well-being is one of the major goals of many individuals worldwide. In pursuit of this goal and in recent years, the Positive Psychology (PP) [14] movement has gained interests internationally for increasing awareness and empirical research on the importance of positive emotions, character strengths, and positive psychological dispositions (i.e., positive traits). This emerging field is meant to complement and work alongside our existing knowledge of psychopathology such as addictions. Investigations in this field are interested in protective factors and individual traits that prevent the development of disorders, as well as strengths that develop despite hardships that increase future psychological prognosis and recovery. The integration of these positive concepts into mainstream psychopathology such as PG is still at its infancy, especially among Chinese populations. The current study focuses on the predictive ability of positive psychological dispositions or traits on PG among the Chinese.

Chinese individuals were selected as the focus of this study for various reasons. In 1998 there were approximately 1.3 billion Chinese worldwide, and while the vast majority lived in mainland China, 37 million lived elsewhere [15]. Representing 22% of the planet's population, Chinese people are the largest ethnic group in the world [16]. It is also not uncommon to encounter anecdotal media coverage on pathological gambling among Chinese individuals with speculations of prostitution and drug-dealing to repay debts, and parental neglect of young children stemming from gambling addiction [17]. Moreover, empirical evidence of PG among the Chinese does suggest that gambling is a popular recreational activity and prevalence rates are higher in this population in comparison with Western populations [9], [17]–[19]. Gambling remains popular among Chinese Diaspora due to the fact that it is an acceptable form of social activity in the community [4], [20], [21]. Therefore, more research on PG among the Chinese is needed for a better understanding of factors that contribute to PG, especially research on positive traits that influence PG severity due to its scarcity in the literature.

One of the main goals of research and treatment in gambling is the actualisation and attainment of the full potential of recovered gamblers. With this goal in mind, there is an important avenue for knowledge development in the intertwining of both positive traits and negative determinants of PG. In essence, gambling treatment and empirical research are not just based on fixing problems, but also on building strengths and developing social, intellectual, and emotional well-being. The integration of Positive Psychology (PP) into developmental psychopathology [22], population research [23], and oppositional defiant disorder [24] were conducted with much success. Furthermore, low positive affect is related to depression and negative symptoms of schizophrenia [25]. It was argued that the best way to merge PP and psychopathology research is by gradually infusing PP into current psychopathology research [26]. Hence, strength-based empirical investigation of problem gambling proposed in this study is an attempt to bridge the gap in literature. The next section elaborates on PP character strengths under investigation and rationale for including them.

### Positive Psychology (PP) character strengths or positive traits (independent variables)

*Curiosity* as a character strength or positive trait has not been studied empirically in the field of gambling but it has been argued that many gamblers approached the game out of sheer curiosity [27], [28]. Curiosity from a PP perspective relates to autonomy in seeking new experiences and intensity of concentration. Neurological researchers on curiosity and exploratory behaviour with gambling tasks and fMRI scan argued that exploratory behaviour is advantageous from an evolutionary perspective for gathering resources and food [29]. This study also reported that there are specific brain systems responsible for exploratory behaviour. McCown [30] suggested that interventions for PG should acknowledge gamblers' curiosity and channel it through other positive and creative outlets. The predictive ability of curiosity on PG was further examined in this study.

Although there is no research on gambling, the affective state of *gratitude* is reportedly beneficial for the recovery from addictive behaviours [31]. An individual's recovery from an alcoholic identity has been argued to benefit from an outlook of gratitude among other factors such as acceptance and surrender, as overall quality of life gradually improves [32]. The importance of gratitude was also highlighted in a 12-step alcoholic treatment program [33]. Beyond the addiction literature, positive traits such as gratitude can uniquely predict disorders such as depression despite the presence of negative traits and buffer against the development of the disorder [34]. Investigations on the predictive ability of gratitude in gambling behaviour will expand our current knowledge of the development and treatment of PG.

In a study on Thai gamblers, the concept of “distorted” hope was measured with two questions (i.e., “How much do you hope to win any prize,” and “Have you ever won any prize?”) [35]. The authors reported that media reports influence gamblers' superstitious beliefs, which influences their level of hope and in turn influenced their gambling propensity. On the flipside and in other studies, problem gamblers were more likely than non-problem gamblers to gamble for reasons that it gives *hope* for a better future and a possibility to obtain more money [36], [37]. As the levels of hope in its various forms have been found to be important in PG, this study examines the impact of hope on PG. Here, hope is defined as self-reported capability in devising pathways for life goals and agency in thought processes when implementing pathways [38].

Another positive trait that was investigated here is *personal growth initiative*, which has been argued to be an important process of recovery from mental health disorders [39]. This process will benefit clients in other aspects of their lives. For example, individuals who are dissatisfied with their fulfilment at work may actively explore other options to increase fulfilment. In another aspect of personal growth, Ciarrocchi and Reinert [40] measured family environment dynamics among gamblers and found that spouses of gamblers reported higher dissatisfaction for personal growth of gamblers as compared to spouses of non-gamblers. Furthermore, group therapy reported that gamblers enrolled in long-term therapy reported greater satisfaction with *personal growth* dimensions as compared to gamblers enrolled in short-term therapy [40]. In this study, we examine the predictive ability of personal growth initiative on PG.

PG is a form of addiction that is characterised by cognitive preoccupation with gambling and cognitive distortions such as “chasing losses.” On the flipside, *mindfulness* or *mindful attention* is characterised by conscious attention and awareness of the antecedents of existing behaviour [41]. Mindfulness was linked with less severe gambling problems and problem gamblers were reported to be less mindful of the present [41]. Research on cognitive-behavioural treatment of gambling has reported that mindful meditation is effective in reducing anxiety and depression, and helps problem gamblers cope with gambling-related cognitive distortions [42], [43]. In this study, we investigate the impact of mindfulness on PG among the Chinese. The next section elaborates on the dependent variables in focus.

### Measurements of problem gambling and other dependent variables

Utilizing valid screening tools for Chinese PG is essential in the advancement of our understanding of PG among the Chinese. Several evidence-based instruments such as the South Oaks Gambling Screen (SOGS) [44], DSM criteria [45], [46], Problem Gambling Severity Index (PGSI) [47] that is derived from the Canadian Problem Gambling Index (CPGI), and Victorian Gambling Screen [48] have been developed to measure problem and pathological gambling, and also most recently renamed as “Gambling Disorder.” The predecessors of PGSI such as SOGS have been used widely in gambling research and validated among various population groups from countries such as Hong Kong and Singapore [49]–[51]. Hence, the current study uses SOGS as a measure of pathological gambling to provide a consistent backdrop from which past research on gambling correlates can be compared. However, as recent evidences suggest that PGSI demonstrated the most valid and reliable psychometric properties in measuring PG prevalence among Western populations [52], we utilise PGSI as a newer and improved measurement of PG among the community in hopes of providing future research with a compatible comparison. Furthermore, psychometric evaluations among Chinese individuals provide support for good scale reliability and validity [53].

Equally as important as measures of pathological gambling and PG are measures of gambling correlates that predict gambling behaviour. Research suggests that gambling-related cognitions such as erroneous beliefs, expectancies, illusion of control and perpetuating gambling thoughts play a crucial role in the development and maintenance of gambling behaviour [9], [54], [55]. The influences of gambling urge have also been considered as an important factor in the development of PG [10], [56], [57]. Although previous studies discussed the role of gambling cognitions and urges in the development of gambling problems, we have little understanding of variables that can predict *both* gambling cognitions and urges. Hence, this study investigates the predictive ability of positive traits on the factors that are clearly important to the development of PG—gambling-related cognitions and urges.

### Rationale for this study

While the roles of “negative” variables such as depression, anxiety, and stress on PG among the Chinese have been clearly examined [54], [58], there is a scarcity of research evaluating the roles of positive traits or character strengths on PG especially among the Chinese. Expanding from past research, this study attempts to bridge the gap in literature by providing a detailed evaluation of the predictive ability of the positive traits on PG severity, gambling-related cognitions, and gambling urges. The interactions between gender and positive traits on PG will also be detailed in this study. We hypothesized a significant negative (predictive) association between PG-related dependent measures and positive traits such as curiosity, gratitude, hope, personal growth initiative, and mindful attention (e.g., higher level of hope relates to lower PG severity). In other words, we hypothesized that these positive traits predict PG. Based on past research that has consistently found gender differences in PG [9], [17], we hypothesized a moderating effect of gender on these positive traits. An accurate perception of the influence of these factors is important in making informed decisions in treatment provision and development of early intervention strategies that target at-risk individuals that minimize the detrimental effects of PG. Furthermore, a good understanding of these positive traits or character strengths is important in its own right but, just as essential, in future advancement of effective treatment programs among the Chinese that focuses on reducing PG and increasing character strengths.

## Methods

### Ethics statement

“This study has been cleared in accordance with the ethical review processes of the University of Queensland and within the guidelines of the National Statement on Ethical Conduct in Human Research. You are, of course, free to discuss your participation with project staff: Jasmine Loo. If you would like to speak to an officer of the University not involved in the study, you may contact one of the School of Psychology Ethics Review Officers, Dr. John McLean and Dr. Courtney von Hippel. Alternatively, you may contact the University of Queensland Ethics Officer at e-mail: humanethics@research.uq.edu.au.”

Ethical approval and clearance were provided by the respective organisational ethics committee (University of Queensland, Australia and Yung-Ta Institute of Technology and Commerce, Taiwan) and all procedures were carried out according to ethical guidelines. Voluntary participation was followed by an introduction to the research study, explanation on informed consent, and freedom to withdraw participation. No personal identification information was requested and privacy was assured. After obtaining written informed consent, paper and pencil questionnaires were administered individually and participants were thanked and debriefed upon completion.

### Participants

801 Chinese participants from Taiwan were recruited for this study (i.e., Taiwanese Chinese; 52.38% were males and 47.62% were females). All 801 participants were proficient in Chinese language (i.e., Mandarin). The mean age was 25.36 years (*SD* = 10.25) with an age range of 18 to 74 years. Employment categories were the following: 69.50% students, 20.10% in full-time employment, 5.10% in part-time employment, 1.70% were job hunting, 2.50% under disability pension and 1.1.% were retired. Most participants have never married (82.7%), while 15.5% were currently married, 1.1% were separated or divorced, 0.4% were widowed, and 0.3% were in a domestic partnership.

In relation to education, 57.2% of participants have had some college education, 28% completed a Bachelor's degree, 12.3% had up to high school education, and 2.5% completed a Postgraduate degree. Most participants were ancestor worshippers (30.2%), while 23.5% were Buddhists, 22.3% had no religion, 18.4% were Taoists, 4.6% were Catholics or Christians, and 1% believed in other religions. The majority of participants earned less than Taiwan Dollar (**TWD**) 100,000 (73.8%) annually, while 8.1% earned between TWD 100,000–TWD 300,000; 11.5% earned between TWD 300,000–TWD 700,000; 4.5% earned between TWD 500,000–TWD 700,000; and 2.1% earned more than TWD 1,000,000. Using the PGSI cut-offs [45], 42.0% of participants were classified as non-problem gamblers, 21.6% were low-risk gamblers, 27.5% were moderate-risk gamblers, and 8.9% were problem gamblers.

### Measures

Materials consisted of a demographic form and a set of self-report questionnaires. All measures without existing validated Chinese versions (i.e., PGSI) were translated from English to Chinese and back-translated to check for reliability and face validity. A proficient bilingual psychologist and graduate student completed the translations. The scales were revised by two bilingual clinical psychology PhD candidates who were blind to the study to ensure accuracy of translation. Pilot tests on 10 university students were carried out to verify the semantic reliability of each item and to ensure ease of understanding. The preceding steps were then repeated for flagged items after pilot testing. Differences between the versions were discussed thoroughly and edited until a translated version was found to have semantic equality with the original English version.

#### South Oaks Gambling Screen (SOGS) [44]

SOGS is a 20-item self-administered scale for assessing “pathological gambling,” which was established from DSM-III criteria. Reported Cronbach's alpha was 0.97 with the test-retest reliability at 0.71 [44]. Items require yes/no responses and total score ranges from 0 to 20. A score of 0 specifies no problem gambling, 1–4 specifies at-risk gambling behaviour or possible problematic gambling, and a score of 5 or more specifies problem gambling. Cronbach's alpha for the Chinese translation was 0.75 with good construct validity [17].

#### Problem Gambling Severity Index (PGSI) [47]

The PGSI is a 9-item instrument of PG, originating from the 31-item CPGI. Five items of PGSI were derived from SOGS, two items from DSM-IV, and two items were newly developed questions. The 4-point rating scale ranged from “0 – Never” to “3 – Almost always.” A total score of 0 identifies a non-gambler, 1–2 identifies a low-risk gambler, 3–7 identifies a moderate-risk gambler, and 8 or more identifies a problem gambler. Reported Cronbach's alpha was 0.84 with a test-retest reliability of 0.78 [47]. The PGSI has good criterion-related validity when compared with DSM-IV and SOGS, correlating at 0.83 with both measures [47]. Cronbach's alpha for the Chinese translation was reported to be 0.77 with good predictive, concurrent, and discriminant validities [53].

#### Gambling Related Cognitions Scale-Chinese version (GRCS-C) [59], [60]

The GRCS-C is a 23-item scale measuring inaccurate gambling cognitions. There are five subscales in the GRCS-C: (1) GE—Gambling expectancies, (2) IC—Illusion of control, (3) PC—Predictive control, (4) IS—Inability to stop gambling, and (5) IB—Interpretative bias. Responses are measured on a 7-point Likert scale ranging from “1 - *strongly disagree*” to “7 - *strongly agree*” with higher scores indicating more gambling-related cognitive distortions held by the participant. The GRCS-C reported a Cronbach's alpha 0.95 and ranged from 0.83 to 0.89 for the five factors [59]. The GRCS-C also reported good concurrent, predictive, and discriminant validities.

#### Gambling Urge Scale-Chinese version (GUS-C) [57], [61]

This 6-item questionnaire measures gambling urge, which has been found to be important in the maintenance of PG. Participants responded on a 7-point Likert scale “1 - *strongly disagree*” to “7 - *strongly agree*” with higher scores indicating a stronger urge to gamble. The Cronbach's alpha in a Chinese sample was reported to be 0.87 and has adequate concurrent, predictive, and criterion validities [61].

#### Curiosity & Exploration Inventory (CEI-C) [62], [63]

The CEI assesses individual differences in the recognition, pursuit, and integration of novel and challenging experiences and information. The CEI is a 7-item scale and respondents rate items using a 7-point Likert-type scale. The CEI has good internal reliability, and shows moderately large positive relationships with intrinsic motivation, reward sensitivity, openness to experience, and subjective vitality. Cronbach's alphas ranged from .63 to .74 for CEI–Exploration, from .66 to .73 for CEI–Absorption, and from .72 to .80 for CEI–total. Moreover, the CEI has shown incremental validity over and above the overlapping constructs of positive affect and reward sensitivity. The Chinese translation of CEI reported α = 0.68 for the overall scale and has good predictive and criterion validity [63].

#### Gratitude Questionnaire (GQ-C) [64], [65]

The original English language GQ is a 6-item self-report measure (1 = “strongly disagree”, 7 = “strongly agree”) of the disposition to experience gratitude and appreciating the positive in life. Two items are reverse-scored to inhibit response bias. The GQ has good internal reliability, with Cronbach's alphas between .82 and .87, and GQ is positively related to optimism, life satisfaction, hope, forgiveness, empathy and pro-social behaviour, and negatively related to depression, anxiety, materialism and envy. In a Taiwanese validation study, 5-item measure GQ-C was found to be a better fit among the Chinese as compared to the original 6-item measure. Cronbach's alpha was reported to be 0.80 with good construct validity [65]. Hence, the 5-item measure of GQ-C was used in this study.

#### Adult Hope Scale (AHS-C) [66], [67]

The AHS measures Snyder's cognitive model of hope that defines hope as a positive motivational state that is synergistically derived from effective goal-directed energy and pathways in planning to meet goals [37]. The adult hope scale contains 12 items. Four items measure pathways thinking, four items measure agency thinking, and four items are fillers. Participants respond to each item using an 8-point scale ranging from definitely false to definitely true. For the total scale, Cronbach's alphas ranged from .74 to .84. Snyder [38] elaborates on the theory of hope and research implications. When used among Chinese samples, Cronbach's alpha was reported to be 0.70 and the test-retest reliability was 0.86 [67]. The Chinese version evidenced good construct and predictive validity.

#### Personal Growth Initiative Scale (PGI) [39]

The PGI measures a person's active and intentional contribution in evolving and developing as a person (5 minutes to complete). The PGI consists of nine items that are rated on a Likert scale from 1 “Strongly Disagree” to 6 “Strongly Agree”. Item scores are summed to obtain a total PGI score that can range from 0 to 45 (*α* = .78). The PGI is strongly positively related to psychological well-being and negatively related to psychological distress. The scale reported good reliability and validity. In a Mexican sample, Cronbach's alpha was reported to be 0.78 with good construct and predictive validity. The reliability and validity of PGI-Chinese version (PGI-C) was investigated in this study. In a Chinese Taiwanese sample, Cronbach's alpha was reported to be 0.87 with good construct and predictive validity [68].

#### Mindful Attention Awareness Scale (MAAS) [69]

This is a 15-item scale assessing a core characteristic of dispositional mindfulness (i.e., receptive awareness of and attention to what is taking place in the present). Responses are made on a 6-point scale and higher scores represent higher levels of dispositional mindfulness. The MAAS has been validated with university, general population, and cancer patient samples with good psychometric results (*α* = .87). MAAS measures awareness that is predictive of self-regulation and health constructs. In a Chinese sample, Cronbach's alpha was 0.85 with the test-retest reliability at 0.54 and good validity results.

### Procedure

As a part of a larger study, university participants from Taiwan were recruited from universities in Southern Taiwan and Northern Taiwan. Ethical approval and clearance were provided by the respective organisational ethics committee (University of Queensland, Australia and Yung-Ta Institute of Technology and Commerce, Taiwan) and all procedures were carried out according to ethical guidelines. The university participants were recruited from these departments: (1) Nursing, (2) General Education, (3) Mechanical Engineering, (4) Electrical Engineering, (5) Recreation Administration, (6) Business Administration, and (7) Medicine. The community participants were recruited from Southern and Northern Taiwan by word-of mouth and community contacts with companies. Voluntary participation was followed by an introduction to the research study, explanation on informed consent, and freedom to withdraw participation. No personal identification information was requested and privacy was assured. After obtaining written informed consent, paper and pencil questionnaires were administered individually and participants were thanked and debriefed upon completion. All participants were reimbursed with TWD 100.00 (i.e., approximately AUD 4.00) and average time taken to complete the questionnaire was 30 minutes.

## Results

### Preliminary data analysis

All data cleaning and descriptive analyses were conducted using SPSS version 17. Data cleaning included checking accuracy of data entry, missing values, and assumptions of multivariate analysis. All outliers were checked for accurate data entry and were retained as each case is from the intended sample and is a true reflection of the data collected from participants [70]. There were 396 males and 360 females (45 missing data). Missing gender data were not imputed for the same reasons. Non-systematic and minor missing data (less than 5% missing) for all variables were replaced using mean substitution [70]. Four participants' entries were removed due to more than 40% of missing items in each entry. Visual screening of the histogram and statistical tests indicated that there was some univariate kurtosis and skewness. Results of evaluation of assumptions led to transformation of variables to reduce skewness, kurtosis and heterocedasticity. It is important to note that Levene's test can be significant in large samples although group variances are not different and hence, should be interpreted with caution [71]. Data was positively skewed and hence, logarithmic transformation was performed [70]. All analyses were conducted with both non-transformed and transformed data – as no substantive differences were found only the non-transformed results are reported. As shown in **Table 1**, all scales used in this study reported Cronbach's alpha ranging from 0.65 to 0.98, reflecting a range of acceptable to good internal consistency.

**Table 1:** Reliability analyses and correlations between all variables in HMR for each dependent variable.

	SOGS	GRCS	GUS	Gender	CEI-total	CEI-Explore	CEI-Absorb	GQ	AHS-total	AHS-Agency	AHS-Pathway	PGI	MAAS
PGSI (α = .77)	.555**	.421**	.353**	−.135**	.029	.006	.049	−.057	−.055	−.027	−.075*	.018	.002
SOGS		.394**	.330**	−.049	−.009	−.002	−.012	−.046	−.112**	−.110**	−.102**	−.029	−.068
GRCS			.726**	−.077*	.002	−.007	.012	−.162**	−.085*	−.062	−.102**	.011	.063
GUS				−.106**	−.029	−.034	−.015	−.203**	−.092*	−.037	−.144**	.032	.080*
Gender					.029	.085*	−.050	.130**	.055	.045	.052	.041	−.040
CEI-total						.901**	.841**	.359**	.566**	.509**	.544**	.491**	.152**
CEI-Explore							.523**	.341**	.539**	.487**	.513**	.483**	.148**
CEI-Absorb								.278**	.436**	.389**	.431**	.359**	.113**
GQ									.436**	.337**	.486**	.310**	.114**
AHS-total										.939**	.917**	.692**	.238**
AHS-Agency											.723**	.691**	.226**
AHS-Pathway												.588**	.218**
PGI													.347**
Cronbach's α	0.83	0.98	0.94	–	0.77	0.71	0.65	0.76	0.85	0.85	0.80	0.92	0.87

*Note*: PGSI = Problem Gambling Severity Index, GRCS = Gambling-Related Cognitions Scale, GUS = Gambling Urge Scale, CEI = Curiosity Exploration Inventory, GQ = Gratitude Questionnaire, AHS = Adult Hope Scale (i.e., Hope Scale), PGI = Personal Growth Initiative Scale, MAAS = Mindful Attention Awareness Scale.

* *p*<0.05.

** *p*<0.01.

#### Zero-order correlations and results of the hierarchical multiple regression (HMR) assessing the effects of positive psychological dispositions and interactions with gender on outcome variables

Examination of the linear relationships between socio-demographic variables (i.e., age, gender, marital status, and employment) and PG showed significant correlations between PG and gender; hence, gender effect was controlled for in the analysis. **Table 1** displays the reliability analyses and correlations between the independent variables and dependent variables. AHS-Pathway was significantly negatively correlated with problem gambling (PGSI score). AHS-total, AHS-agency, and AHS-pathway showed significant negative correlations with SOGS. GQ, AHS-total, and AHS-pathway showed significant negative correlation with GRCS; while GQ, AHS-total, AHS-pathway, and MAAS showed significant negative correlation with GUS.

Preliminary steps were taken in all analyses to check for adherence to assumptions. To reduce problems associated with multicollinearity, all independent variables and moderator variables were centred (i.e., standardized) [72], [73]. Centred variables were created by subtracting the mean value from the variable while the interaction variable was created by multiplying the two mean centred independent and moderator variables together [72]. A moderation effect was considered evident only when the interaction term (e.g., gender x PGI total) in the regression was significant. With the use of a *p*<.001 criterion (i.e., values larger than 31.264, *df* = 11) for Mahalanobis distance [70], no outliers among the cases were identified. Using the variation inflation factor (VIF), multi-collinearity was checked and all variables reported values below 10 [71], which indicate that the data did not violate the assumption of multi-collinearity. No suppressor variables were found. **Table 2** tabulates the means and standard deviations for all variables.

**Table 2:** Means and standard deviations for each scale and subscale (Chinese version).

Variable	Total^A^	Mean	Standard Deviation
South Oaks Gambling Scale (SOGS-C)	19.00	1.84	2.64
Problem Gambling Severity Index (PGSI-C)	27.00	2.55	3.75
Gambling Cognitions total (GRCS-C)	161.00	41.83	26.85
Gambling Urge (GUS-C)	42.00	9.70	7.04
Curiosity & Exploration total (CEI-C)	49.00	32.03	6.11
CEI-Exploration	28.00	18.33	3.88
CEI-Absorption	21.00	13.70	3.11
Gratitude (GQ-C)	42.00	31.46	5.78
Hope (AHS-C)	96.00	43.80	8.63
AHS-Agency	48.00	20.65	4.98
AHS-Pathway	48.00	23.11	4.31
Personal Growth Initiative (PGI-C)	54.00	36.80	7.81
Mindful Attention Awareness (MAAS-C)	90.00	58.50	10.94

*Note*: GRCS = Gambling Related Cognitions Scale, CEI = Curiosity Exploration Inventory, GQ = Gratitude Questionnaire, AHS = Adult Hope Scale (i.e., Hope Scale), MAAS = Mindful Attention Awareness Scale.

A = Highest total score possible for that scale.

A series of Hierarchical Multiple Regression (HMR) analyses were conducted in this order: (1) SOGS-C as DV with total IV scale scores, (2) SOGS-C as DV sith IV subscale scores, (3) PGSI-C as DV with total IV scale scores, (4) PGSI-C as DV with IV subscale scores, (5) GRCS-C as DV with total IV scores, (6) GRCS-C as DV with IV subscale scores, (7) GUS-C as DV with total IV scores, and (8) GUS-C as DV with IV subscale scores.

**Figure 1:**
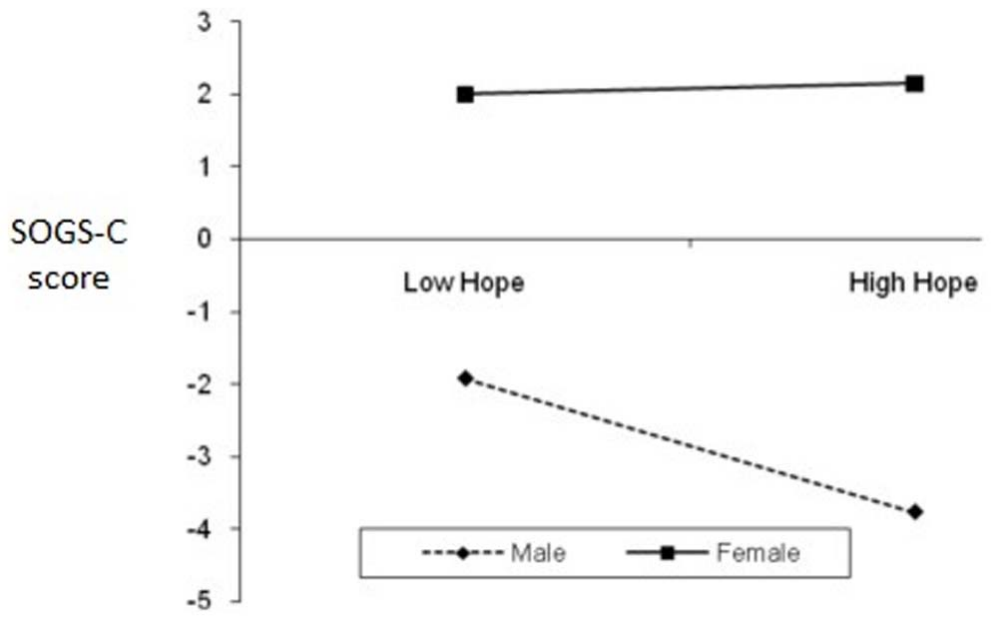
The interaction of gender and hope (AHS-total) on SOGS-C.

First, HMR was used to assess the extent to which these variables could predict PG. South Oaks Gambling Scale (SOGS-C) total score was used as the dependent variable (**DV**). The independent variables (**IV**) and interaction variables were: (Step 1) Gender, (Step 2) CEI-total, GQ-total, AHS-total, PGI-total and MAAS-total, and (Step 3) Two-way interactions between gender and total scores entered in Step 2. **Table 3** displays the standardized regression coefficients (*β*), *R^2^* change, *R*, *R^2^*, and Adjusted *R^2^* after entry of all independent variables to predict outcome variables. *R* was significantly different from zero at Step 2 and Step 3. HMR results showed that the overall model was significant *F* (11, 744) = 3.14, *p*<.001. The *R^2^* value of 0.044 indicates that 4.4% of the variability in SOGS-C is accounted for by the predictors. Only these variables significantly predicted and accounted for variance in SOGS-C scores: (1) CEI-total (accounted for 0.03% of variance), (2) AHS- total (0.80%), (3) MAAS-total (0.50%), and (4) Interaction between gender and AHS-total (1.70%). **Figure 1** shows the simple slopes of the level of hope (AHS-total) for each group of gender (i.e., moderator). The link between hope and SOGS-C is significant for males, but not for females. For males, higher levels of hope predict lower SOGS-C score.

**Table 3:** Results of the hierarchical multiple regression (HMR) assessing the effects of positive psychological dispositions and interactions with gender on outcome variables (**Total scores**).

Predictors	SOGS-C		PGSI-C		GRCS-C		GUS-C	
	Δ*R* ^2^	*β* a	Δ*R* ^2^	*β* a	Δ*R* ^2^	*β* a	Δ*R* ^2^	*β* a
***Step 1***	.002		.018***		.006*		.011**	
Gender^b^		−.049		−.135***		−.077*		−.106**
***Step 2***	.021**		.013		.039***		.062***	
CEI-total		.089*		.042		.051		.023
GQ-total		−.001		−.056		−.157***		−.217***
AHS-total		−.179***		−.063		−.126*		−.104*
PGI-total		.089		.122**		.116*		.165***
MAAS-total		−.074*		−.050		.056		.063
***Step 3***	.021**		.007		.005		.009	
Gender×CEI		−.066		−.005		−.033		.017
Gender×GQ		−.040		.030		.014		.048
Gender×AHS		.187***		.041		.071		.081
Gender×PGI		−.042		−.061		.015		−.061
Gender×MAAS		.052		.075*		−.007		−.016
Total *R*	0.211***		0.195**		0.223***		0.287***	
Total *R* ^2^	0.044***		0.038**		0.050***		0.082***	
Adjusted *R^2^*	0.030***		0.024**		0.036***		0.069***	
*F* (11, 744) =	3.14***		2.67**		3.55***		6.05***	

*Note:*

a Standardized beta weights at entry.

b Gender: −1 = Male, 1 = Female.

* *p*<0.05.

** *p*<0.01.

*** *p*<0.001.

**Figure 2:**
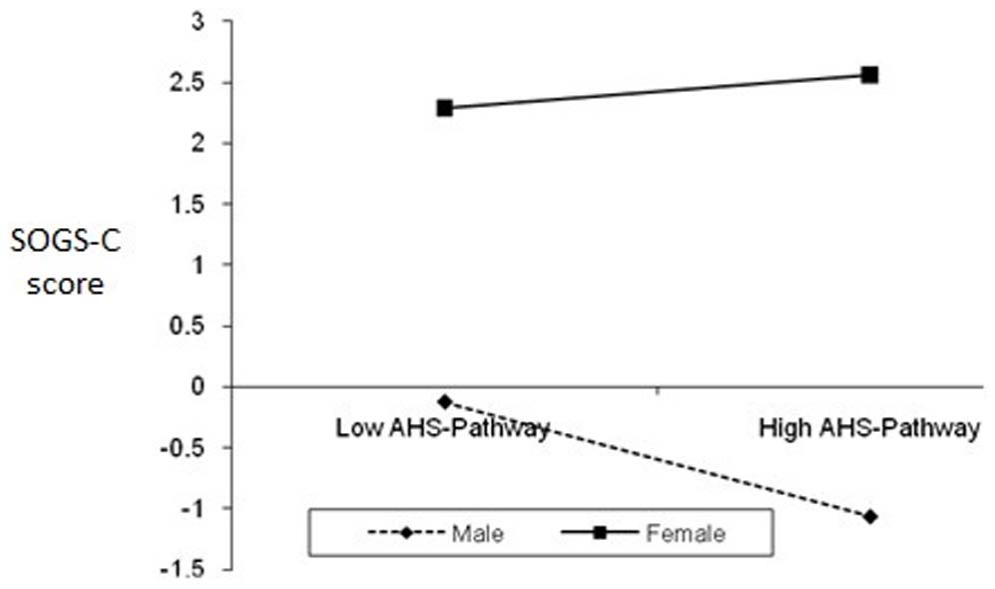
The interaction of gender and AHS-Pathway on SOGS-C.

Second, HMR analysis was performed to assess the extent to which the subscales and total score (if single-factor structure) could predict SOGS-C. SOGS-C total score was used as the dependent variable (**DV**). The independent variables (**IV**) and interaction variables were: (Step 1) Gender, (Step 2) CEI-Exploration, CEI-Absorption, GQ-total, AHS-Agency, AHS-Pathway, PGI-total and MAAS-total, and (Step 3) Two-way interactions between gender and variables entered in Step 2. **Table 4** displays the standardized regression coefficients (*β*), *R^2^* change, *R*, *R^2^*, and Adjusted *R^2^* after entry of all independent variables (subscales included) to predict outcome variables. *R* was significantly different from zero at Step 2 and Step 3. HMR results showed that the overall model was significant *F* (15, 740) = 2.38, *p* = .002. The *R^2^* value of 0.046 indicates that 4.6% of the variability in gambling scores is accounted for by the predictors. Only these variables significantly predicted and accounted for variance in SOGS-C scores: (1) AHS-Agency (accounted for 0.81% of variance), (2) MAAS-total (0.50%), and (3) Interaction between gender and AHS-Pathway (1.40%). Similar to the first HMR results, the link between planning to meet goals (AHS-Pathway) and SOGS-C is significant for males, but not for females (see **Figure 2**). For males, higher levels of planning to meet goals predict lower SOGS-C score.

**Table 4:** Results of the hierarchical multiple regression (HMR) assessing the effects of positive psychological dispositions and interactions with gender on outcome variables (**Subscales scores**).

Predictors	SOGS-C		PGSI-C		GRCS-C		GUS-C	
	Δ*R* ^2^	*β* a	Δ*R* ^2^	*β* a	Δ*R* ^2^	*β* a	Δ*R* ^2^	*β* a
***Step 1***	.002		.018***		.006*		.011**	
Gender^b^		−.049		−.135***		−.077*		−.106**
***Step 2***	.023**		.014		.041***		.068***	
CEI-Exploration		.067		.006		−.009		.010
CEI-Absorption		.039		.040		.066		.022
GQ-total		−.006		−.047		−.159***		−.196***
AHS-Agency		−.147**		.017		−.082		.031
AHS-Pathway		−.057		−.082		−.057		−.153**
PGI-total		.099		.113*		.124*		.153**
MAAS-total		−.076*		−.046		.059		.067
***Step 3***	.020*		.010		.006		.015	
Gender×CEI-Exploration		−.061		−.062		−.017		−.054
Gender×CEI-Absorption		−.013		.040		−.024		.055
Gender×GQ-total		−.044		.019		.005		.025
Gender×AHS-Agency		.091		.002		−.005		−.051
Gender×AHS-Pathway		.113*		.057		.084		.138**
Gender×PGI-total		−.041		−.050		.020		−.026
Gender×MAAS-total		.053		.078*		−.012		−.014
Total *R*	0.214**		0.205**		0.230***		0.306***	
Total *R* ^2^	0.046**		0.042**		0.053***		0.094***	
Adjusted *R^2^*	0.027**		0.023**		0.034***		0.075***	
*F* (15, 740) =	2.38**		2.17**		2.75***		5.11***	

*Note:*

a Standardized beta weights at entry.

b Gender: −1 = Male, 1 = Female.

* *p*<0.05.

** *p*<0.01.

*** *p*<0.001.

**Figure 3:**
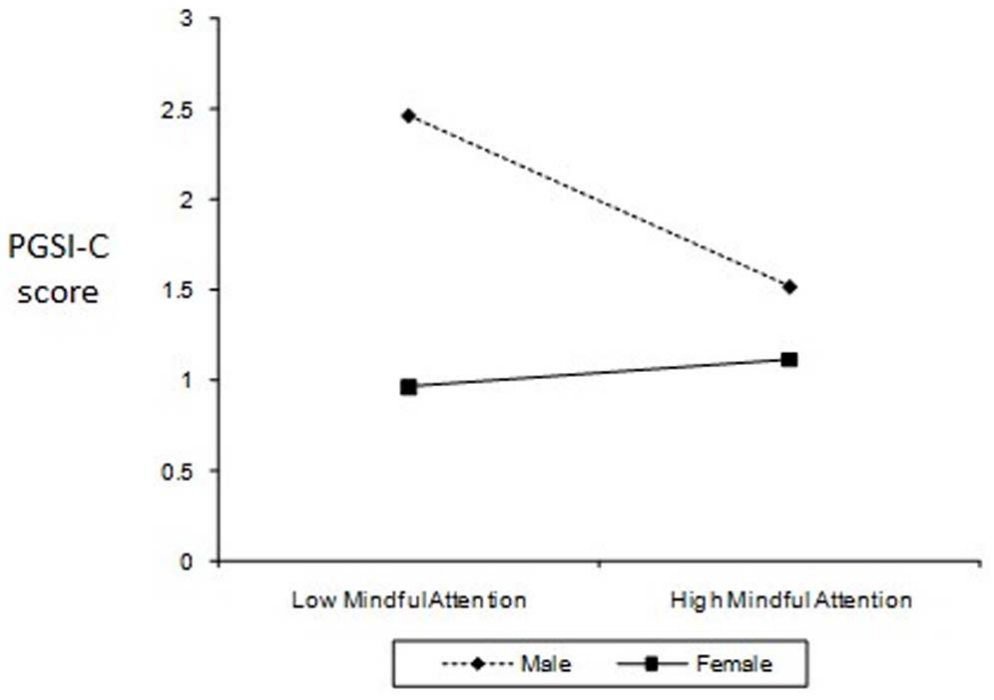
The interaction of gender and mindful attention awareness (MAAS-C) on problem gambling severity (PGSI-C).

In the third HMR, Problem Gambling Severity Index (PGSI-C) total score was used as the dependent variable (**DV**). The independent variables (**IV**) and interaction variables were: (Step 1) Gender, (Step 2) CEI-total, GQ-total, AHS-total, PGI-total and MAAS-total, and (Step 3) Two-way interactions between gender and total scores entered in Step 2. *R* was significantly different from zero at Step 1. As shown in **Table 3**, HMR results showed that the overall model was significant *F* (11, 744) = 2.67, *p* = .002. The *R^2^* value of 0.04 indicates that 4% of the variability in gambling scores is accounted for by the predictors. Only these variables significantly predicted and accounted for variance in PGSI scores: (1) gender (accounted for 1.80% of variance), (2) PGIS- total (1.30%), and (3) Interaction between gender and MAAS-total (0.70%). **Figure 3** shows the simple slopes of the Mindful Attention (MAAS-total) for each group of gender (i.e., moderator). The link between Mindful Attention and PG is significant for males, but not for females. For males, higher Mindful Attention predicts lower PGSI-C score.

The fourth HMR analysis was performed to assess the extent to which the subscales and total score (if single-factor structure) could predict PG. PGSI total score was used as the dependent variable (**DV**). The independent variables (**IV**) and interaction variables were: (Step 1) Gender, (Step 2) CEI-Exploration, CEI-Absorption, GQ-total, AHS-Agency, AHS-Pathway, PGI-total and MAAS-total, and (Step 3) Two-way interactions between gender and variables entered in Step 2. *R* was significantly different from zero at Step 1. As shown in **Table 4**, HMR results showed that the overall model was significant *F* (15, 740) = 2.17, *p* = .006. The *R^2^* value of 0.042 indicates that 4.2% of the variability in gambling scores is accounted for by the predictors. Only these variables significantly predicted and accounted for variance in PGSI scores: (1) gender (accounted for 1.80% of variance), (2) PGIS- total (1.40%), and (3) Interaction between gender and MAAS-total (1%). Similar to the first HMR results, the link between Mindful Attention and problem gambling is significant for males, but not for females (see **Figure 2**).

Fifth, HMR was used to assess the extent to which the positive psychological dispositions could predict gambling-related cognitions. Gambling-Related Cognitions Scale (GRCS-C) total score was used as the dependent variable (**DV**). The independent variables (**IV**) and interaction variables were: (Step 1) Gender, (Step 2) CEI-total, GQ-total, AHS-total, PGI-total and MAAS-total, and (Step 3) Two-way interactions between gender and total scores entered in Step 2. **Table 3** displays the standardized regression coefficients (*β*), *R^2^* change, *R*, *R^2^*, and Adjusted *R^2^* after entry of all independent variables. *R* was significantly different from zero at Step 1 and Step 2. HMR results showed that the overall model was significant *F* (11, 744) = 3.55, *p*<.001. The *R^2^* value of 0.05 indicates that 5% of the variability in GRCS-C is accounted for by the predictors. These variables significantly predicted and accounted for variance in GRCS-C scores: (1) gender (accounted for 0.60% of variance), (2) GQ-total (3%), (3) AHS-total (0.60%), and (4) PGIS- total (0.07%).

Sixth, another HMR analysis was performed to assess the extent to which the subscales and total score (if single-factor structure) could predict gambling-related cognitions. Gambling-Related Cognitions Scale (GRCS-C) total score was used as the dependent variable (**DV**). The independent variables (**IV**) and interaction variables were: (Step 1) Gender, (Step 2) CEI-Exploration, CEI-Absorption, GQ-total, AHS-Agency, AHS-Pathway, PGI-total and MAAS-total, and (Step 3) Two-way interactions between gender and variables entered in Step 2. *R* was significantly different from zero at Step 1 and Step 2. As shown in **Table 4**, the overall HMR model was significant *F* (15, 740) = 2.75, *p*<.001. The *R^2^* value of 0.053 indicates that 5.3% of the variability in GRCS-C is accounted for by the predictors. These variables significantly predicted and accounted for variance in GRCS-C scores: (1) gender (accounted for 0.60% of variance), (2) GQ-total (2.5%), and (4) PGIS- total (0.07%).

The seventh HMR was used to assess the extent to which the positive psychological dispositions could predict gambling urges. Gambling Urge Scale (GUS-C) total score was used as the dependent variable (**DV**). The independent variables (**IV**) and interaction variables were the same as the first and third HMR. *R* was significantly different from zero at Step 1 and Step 2. As shown in **Table 3**, the overall model was significant *F* (11, 744) = 6.05, *p*<.001. The *R^2^* value of 0.08 indicates that 8% of the variability in GUS-C is accounted for by the predictors. These variables significantly predicted and accounted for variance in GUS-C scores: (1) gender (accounted for 1.12% of variance), (2) GQ-total (4.20%), and (3) PGIS- total (0.36%).

**Figure 4:**
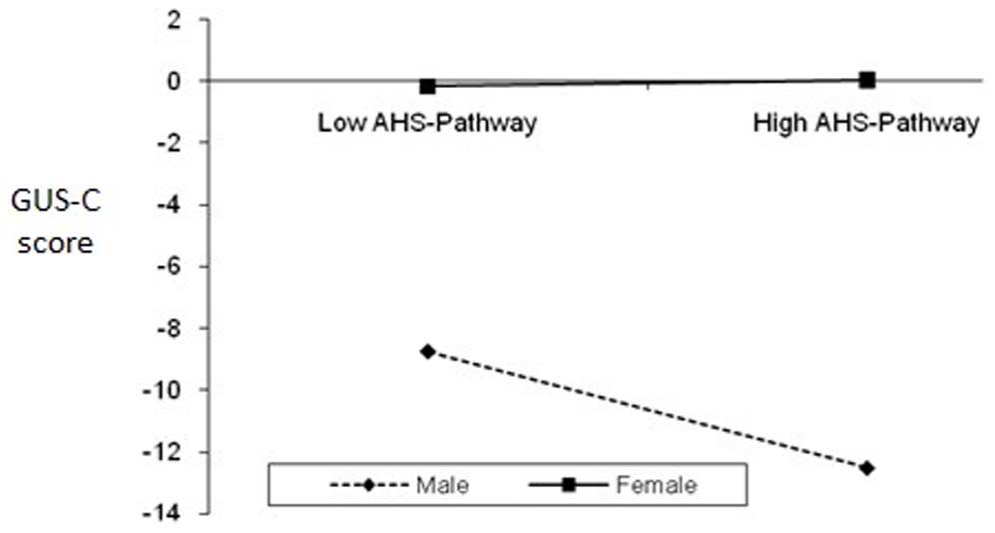
The interaction of gender and successful planning to meet goals (AHS-Pathway) on Gambling Urges (GUS-C).

The eighth HMR was used to assess the extent to which the positive psychological dispositions subscales and total score (if single-factor structure) could predict gambling urges. Gambling Urge Scale (GUS-C) total score was used as the dependent variable (**DV**). The independent variables (**IV**) and interaction variables were the same as the second and fourth HMR. *R* was significantly different from zero at Step 1 and Step 2. As shown in **Table 4**, the overall HMR model was significant *F* (15, 740) = 5.00, *p*<.001. The *R^2^* value of 0.094 indicates that 9.4% of the variability in GUS-C is accounted for by the predictors. These variables significantly predicted and accounted for variance in GUS-C scores: (1) gender (accounted for 1.12% of variance), (2) GQ-total (4.20%), (3) AHS-Pathway (1.30%), (4) PGIS- total (0.36%), and (5) Interaction between gender and AHS-Pathway (1.40%). **Figure 4** shows the simple slopes of the successful planning to meet goals (AHS-Pathway) for each group of gender (i.e., moderator). The link between AHS-Pathway and GUS-C is significant for males, but not for females. For males, more successful planning to meet goals predicts lower gambling urge score.

## Discussion

This study examined the ability of positive traits such as curiosity, gratitude, hope, personal growth initiative, and mindfulness to predict PG among Chinese individuals residing in Taiwan. We hypothesized that these positive traits or character strengths predicts PG (i.e., higher scores on these positive traits will predict lower PG-related scores). Partially supporting our hypotheses, the results revealed that certain positive traits predict PG (PGSI score), pathological gambling (SOGS score), gambling-related cognitions (GRCS), and gambling urges (GUS).

The results showed significant negative correlation (small effect size) between PGSI and hope-pathway (i.e., planning to meet goals). In other words, higher PGSI score was related to lower hope-pathway. Hope was negatively related to SOGS, GRCS, and GUS; although significant, the effect sizes are small. Gratitude was significantly negatively related to GRCS and GUS. Contrary to predictions, mindfulness was positively correlated with GUS; however, the effect size is small. Regression analyses on the total scores indicated that CEI-total, AHS-total, and MAAS-total scores significantly predicted SOGS scores in this Taiwanese Chinese sample (i.e., higher scores on these positive traits predicted lower SOGS). However, only PGI-total (personal growth initiative) significantly predicted PGSI scores. GQ-total, AHS-total, and PGI-total significantly predicted both GRCS and GUS. These findings provide some support for past research that has found that curiosity [30], hope [36], mindfulness [41], gratitude [34], and personal growth initiative [40] play a role in the development and recovery of psychopathology.

In this study, we found that these positive traits play a role in the development of PG and related variables such as gambling-related cognitions or gambling urges. However, the direction of predictive ability for PGI-total is contrary to our initial hypotheses, as higher PGI-total scores predicted *higher* PGSI score, GRCS, and GUS. One plausible explanation is: although personal-growth initiative is important for the process of recovery in psychopathology [74], its effects on the development of PG is different in nature. Similar findings were obtained in a study on college students where unusually positive mood characterized by positive urgency was identified as a significant predictor of higher gambling propensity [75]. Perhaps, individuals with higher PGI scores used gambling as an outlet for “personal growth,” as it is common for Chinese individuals to link monetary gains with personal success in life [9]. Also, PG scores in this sample may not be as serious as compared to clinical populations; hence, there is a possibility that results may change according to the severity of PG.

Although the correlation between PGSI and SOGS was significant at *r* = 0.55 and PGSI was able to predict SOGS [53], different positive traits predicted these PG measures. This discrepancy may be linked to criticisms of the use of SOGS in community prevalence studies because SOGS was originally developed for clinical use and it tended to overestimate PG prevalence [52], [76]. Hence, the differences in total PG score may contribute to different regression results. Furthermore, SOGS contain more questions regarding money problems than PGSI. Perhaps, curiosity, hope, and mindfulness play a significant role in the development of issues with money.

HMR analyses on subscales revealed that goal-directed energy (AHS-Agency) was a significant predictor of SOGS. None other positive traits subscales significantly predicted PGSI and GRCS scores. However, planning to meet goals (AHS-Pathway) significantly predicted GUS. As AHS-total score was a significant predictor of both SOGS and GUS, these results are not surprising and provide some support for past research [35]. Higher levels of goal-directed energy predicts lower SOGS score, while more plans directed toward meeting goals predicted lower gambling urges. Among the positive traits investigated here, curiosity did not significantly predict PG or gambling correlates. Perhaps, curiosity and exploratory behaviour benefit activities in daily life [29], but does not necessarily impact on gambling behaviour.

Partially supporting our predictions and past research [9], [17], significantf gender interaction effects were evident for overall level of hope and planning to meet goals (AHS-Pathway) in predicting SOGS (i.e., gender moderated the effect of these variables in predicting SOGS). Gender moderated the effect of mindfulness (MAAS) in predicting PGSI, while gender moderated the effect of AHS-pathway on GUS. The link between these positive traits and PG is significant for males, but not for females. For example, higher mindfulness predicted lower PGSI score only among male participants. A possible explanation is because females are generally hopeful and mindful; hence no differences are seen between low-high hope and mindful female groups. However, males can be distinguished between low-high groups on these PP traits and higher levels of such positive traits then predicted lower GUS or PGSI. Differences in results related to PGSI and SOGS can be attributed to the nature of these scales where PGSI is a 12-month measure while SOGS is a lifetime measure. As noted in the Method section, a strong correlation between PGSI and SOGS is partly attributed to some PGSI items being derived originally from SOGS items.

All findings should be interpreted in light of the limitations of this study. The predictive abilities of most variables were small and run the risk of being essentially meaningless, as indicated by the *R^2^* value and these might not be detected given a smaller sample size. The participants were recruited using convenience sampling method as opposed to random sampling (e.g., using census data) where every member of the population has an equal opportunity of being selected. Such research will require national collaborative effort and significant funding. Hence, the current study provided a good start on descriptive and inferential analysis of patterns of PP and PG among the Chinese. As with all survey research, we relied on self-reported PG involvement, which is dependent on demand characteristics and recall bias. It will be interesting to examine third-party estimates of PG and to simulate an experimental gambling test that will accurately measure actual gambling behaviour while manipulating positive psychological dispositions. Future research will also benefit from investigating other positive traits such as optimism and grit. Theoretical investigations via structural equation modelling will be an interesting method of combining both negative and positive traits in the prediction of PG. Longitudinal studies should be conducted to establish causal links between the predictor variables and problem gambling [75].

These findings have essential implications in our knowledge and treatment of PG among the Chinese. The positive traits or character strengths reported above are factors that should be addressed by mental health professionals in preventive and treatment programs among Chinese individuals. Results reported here provided some support for our hypotheses and strengthen the integrative efforts on Positive Psychology and PG. Higher gratitude and hope was found to predict lower PG, gambling-related cognitions, and gambling urges. Meanwhile, higher mindfulness predicted lower PG, but only among Chinese males. Contrary to predictions, lower personal growth initiative predicted lower PG, gambling-related cognitions, and gambling urges. Future interventions will benefit from improving the client's level of gratitude, hope, and mindfulness; while individuals higher in personal-growth initiative may benefit from rechannelling this positive character strength via constructive outlets such as improving professional competency in the workplace that contributes inevitably to higher monetary reward. As the field of integrative research between positive traits and PG is still at its infancy, the questions for further investigations are vast and varied, but this study created a platform from which future inquiries can be formulated.
